# Simultaneous Quantification of Antipsychotic and Antiepileptic Drugs and Their Metabolites in Human Saliva Using UHPLC-DAD

**DOI:** 10.3390/molecules24162953

**Published:** 2019-08-14

**Authors:** Ewelina Dziurkowska, Marek Wesolowski

**Affiliations:** Department of Analytical Chemistry, Medical University of Gdansk, Gen. J. Hallera 107, 80-416 Gdansk, Poland

**Keywords:** saliva, simultaneous quantification, neuroleptics, carbamazepine, metabolites, UHPLC-DAD

## Abstract

Neuroleptics and antiepileptics are excreted in saliva, which can, therefore, be very useful in determining their concentration in the body. This study presents a method developed to simultaneously identify five neuroleptics—olanzapine, quetiapine, risperidone, aripiprazole, and clozapine—and the antiepileptic carbamazepine together with their metabolites: N-demethyl olanzapine, norquetiapine, 9-OH-risperidone, dehydroaripiprazole, N-desmethylclozapine, and carbamazepine-10,11 epoxide. Chlordiazepoxide was used as the internal standard. Strata-X-C columns were used for isolation of the compounds. Chromatographic analysis was carried out using UHPLC with a diode array detector (DAD). A mixture of acetonitrile and water with the addition of formic acid and 0.1% triethylamine was used as the mobile phase. The developed method was validated by determining the linearity for all analytes in the range 10–1000 ng/mL and the value of *R*^2^ > 0.99. Intra- and inter-day precision were also determined, and the relative standard deviation (RSD) value in both cases did not exceed 15%. To determine the usefulness of the developed method, saliva samples were collected from 40 people of both sexes treated with the tested active substances both in monotherapy and in polypragmasy. In all cases, the active substances tested were identified.

## 1. Introduction

Antipsychotic drugs, also known as neuroleptics, are most often used in schizophrenia, one of the most debilitating and serious psychiatric diseases. Neuroleptics do not cure the disease but allow the functioning of an affected person in society by inhibiting β-adrenergic receptors, as well as muscarinic and histamine receptors. Second-generation drugs also block serotonin receptors [1]. In addition to typical use, antipsychotics are also used to treat organic depression, mixed addictions, schizoaffective disorder, and bipolar disorder. Due to the many side effects, they are mostly used individually. When the potential of their neuroleptic effect is required, they are prescribed in polypragmasy or together with carbamazepine, which also stabilizes the mood. The combination of neuroleptics is also indicated in the case of severely intensified metabolic symptoms, such as weight gain occurring while using olanzapine, salivation, or dry mouth, one of the most common side effects of clozapine. In both cases, the simultaneous use of aripiprazole reduces the severity of side effects [2].

Saliva can be a convenient biological material for testing the concentration of biologically active substances in the body. Only the non-protein fractions of the drugs can be determined in saliva. This property is particularly important in the case of compounds that strongly bind to blood proteins. Due to the differences among individuals in blood and saliva pH, as well as drug pK, some substances, primarily alkaline, may have a greater affinity for saliva. Acidic drugs enter the saliva to a lesser extent [3].

Among the drugs analyzed in saliva, the most common are antiepileptic preparations, which have a high correlation between their blood and saliva levels [4,5,6,7]. For example, saliva has been proven useful in monitoring therapy using carbamazepine. The potential of saliva as a biological material to monitor the concentration of drugs in the body was also determined for antipsychotics, namely, quetiapine [8,9,10], clozapine [10,11], and risperidone [10,12,13,14,15]. The data in the literature indicate that analytes are usually determined using liquid chromatography with UV detection [4,5,11,15], mass spectrometry [8,9,10,12,13] or coulometry [14].

Saliva sampling is not difficult. However, it should be noted that the sample should be frozen as soon as possible to avoid drug decomposition and excessive bacterial growth. If it is impossible to freeze the sample immediately, it is recommended to store it in the refrigerator. Saliva samples containing neuroleptics or antiepileptics are most frequently purified using liquid–liquid extraction (LLE) [4,10,11,16]. Other methods of sample purification include solid-phase extraction (SPE) [13], microextraction by packed sorbent (MEPS) [14,15], and precipitation [5,12]. The SPE method is one of the most often chosen for the isolation of analytes because of its selectivity and high extraction recovery. Solid-phase extraction enables the isolation of compounds with different structures and physicochemical properties, both polar and non-polar. The commercial availability of many types of sorbents allows for the appropriate selectivity of the extraction process. In addition, it is also possible to control and modify each stage of the SPE. It includes modification of the sample, such as dilution or change in pH, the choice of cartridge, the solutions used to wash it, and the solvent mixture used during analyte elution from the sorbent. Moreover, it can be easily automated, which is particularly important for routine control of the level of the drug in the body. In many laboratories, where several dozens of samples are analyzed daily, SPE is the most common method of analyte isolation [17,18].

**Figure 1:**
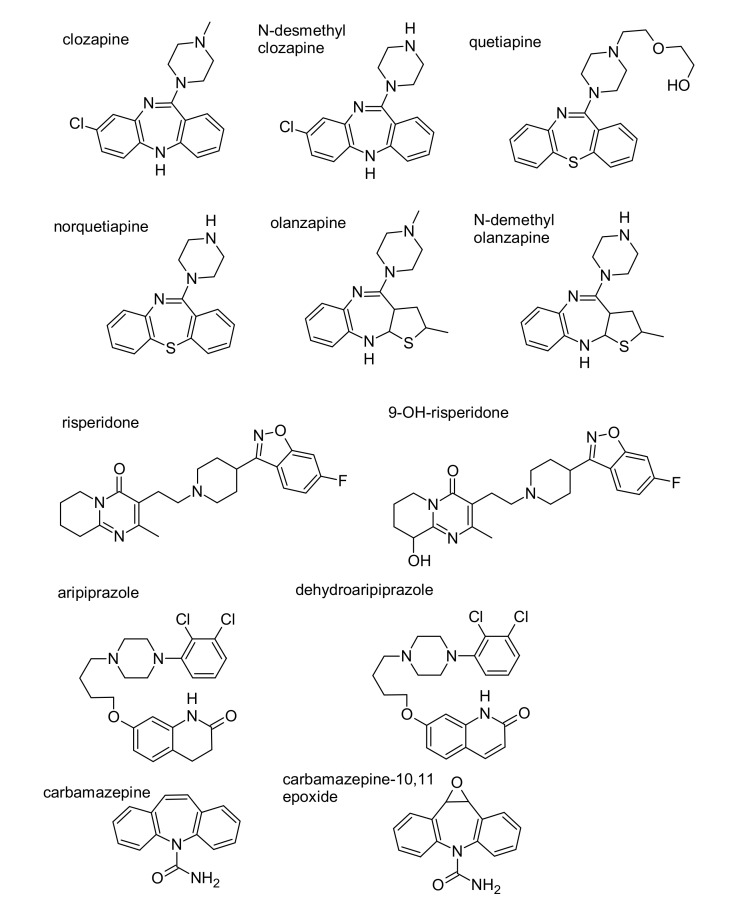
Chemical formulas of the analyzed substances and their metabolites.

A lack of response to treatment with antipsychotics and a deterioration in the mental health of the patient are absolute indications to determine the frequency of drug intake. In the case of discontinuation of treatment, determination of the drug should be accompanied by determination of its metabolites. The frequent use of a combination of two neuroleptics combined with carbamazepine to potentiate their action means that a quick and sensitive method is needed to monitor the patient’s medication intake. Considering the above, the aim of this study was to develop a fast, sensitive, and selective method to detect the presence of five neuroleptics in saliva (clozapine, quetiapine, olanzapine, risperidone, and aripiprazole) together with their metabolites (N-desmethylclozapine, norquetiapine, N-demethyl olanzapine, 9-OH-risperidone, and dehydroaripiprazole) as well as carbamazepine and carbamazepine-10,11 epoxide (Figure 1). Solid-phase extraction is applied for the isolation of analytes. The literature data show that this method has not been developed to date.

## 2. Results and Discussion

### 2.1. Chromatographic Separation

**Figure 2:**
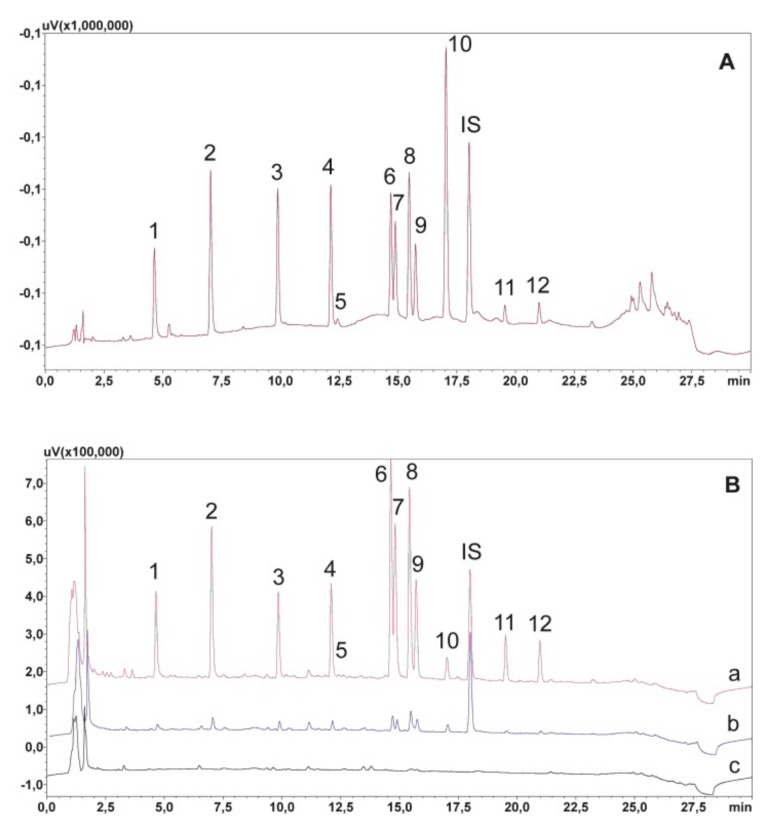
(**A**) Chromatogram of the standard solutions obtained by optimized UHPLC; (**B**) chromatogram of saliva sample extracts obtained after washing the sorbent with water and a mixture of water and methanol (1:1) and eluted with 5% ammonia solution in methanol; a—spiked with 750 ng/mL; b—spiked with 150 ng/mL; c—blank saliva sample extract. 1—N-demethyl olanzapine; 2—olanzapine; 3—9-OH-risperidone; 4—risperidone; 5—carbamazepine-10,11 epoxide; 6—N-desmethylclozapine; 7—norquetiapine; 8—clozapine; 9—quetiapine; 10—carbamazepine; IS—chlordiazepoxide; 11—dehydroaripiprazole; 12—aripiprazole.

The first element of the method optimized to allow the simultaneous determination of analytes was the selection of the chromatographic separation parameters. Some of the compounds tested have similar physicochemical properties due to the fact of their similar structure. This was especially evident in the case of clozapine, quetiapine, and their metabolites N-desmethylclozapine and norquetiapine. In addition, risperidone and carbamazepine-10,11 epoxide also have very similar retention times. The precise separation of all analyzed compounds extended the time of the linear increase in the concentration of solvent B with the following gradient: 0–21 min, 15–40%; 21–23 min, 40–65%; 23–25 min, 65–90%; 25–30, 90–15%; and the mobile phase flow reduced to 0.6 mL/min. The next step was to select the appropriate wavelength for observing the chromatographic separation. The best was 240 nm, where the peak areas of olanzapine, risperidone, clozapine, quetiapine, and carbamazepine together with their metabolites were analyzed. In contrast, aripiprazole and dehydroaripiprazoles were detected at 214 nm. Chlordiazepoxide was used as an internal standard for UV detection. Because chlordiazepoxide is a benzodiazepine that has not been used in medicine for many years, there is no possibility of any accidental presence of this substance in the patient’s saliva. Moreover, chlordiazepoxide is characterized by good absorbance at both 240 and 214 nm and was, therefore, observed at both wavelengths. A chromatogram of the standard solutions obtained by optimized UHPLC is presented in Figure 2A.

### 2.2. Extraction Procedure

To optimize the extraction process, the effects of pH and the SPE sorbent type on the purification of samples were investigated. A detailed description of the optimization of the extraction procedure has been published previously [19]. Briefly, three types of SPE columns were selected for the investigation: Strata-X, Strata-X-C, and Strata-X-CW. The best purification of the extracts and highest recovery of almost all analytes at low limit of quantification (LOQ) values were achieved when Strata-X-C columns were used. When testing the effect of pH on the dilution of the sample, 2% formic acid (FA) and a mixture of methanol and water (1:1) were added to 1 mL of defrosted saliva. The most efficient washing process was achieved when sorbents were washed with water and then with a mixture of water with methanol (1:1). The analytes were eluted with a 5% solution of ammonia in methanol. Figure 2B presents the chromatogram of the extracts of blank and spiked saliva with an appropriate amount of analyte mixture (150 and 750 ng/mL) obtained after using the final extraction procedure.

### 2.3. Method Validation

To avoid excessive dilution of the sample and to eliminate errors resulting from repeated dosing of various volumes of analyte solutions, working solutions were prepared in the form of a mixture with the same concentration of all analyzed compounds (1 or 10 μg/mL). Calibration for all analytes was determined in the concentration range of 10–1000 ng/mL. Limit of quantification was calculated based on the procedure presented in Section 3.5.4. The LOQs for individual analytes were in the range of 3–10 ng/mL. In view of that, the regulatory guidance does not permit extrapolation of concentrations below the lowest calibrator point, the concentration of 10 ng/mL was assumed as the LOQ. In all cases, the method was linear (*R*^2^ ≥ 0.99). Detailed data are presented in Table 1. The obtained results indicate that the method is precise, as confirmed by an RSD value below 15% for each of the analytes, both for intra- and inter-day precision.

### 2.4. Extraction and Absolute Recovery

The extraction recovery was determined for two analyte concentrations (150 ng/mL and 750 ng/mL), comparing the peak areas of analytes extracted using the developed method with the peak areas of blank post-spiked samples. The extraction method was suitable for all analyzed compounds except carbamazepine-10,11 epoxide, for which the recovery did not exceed 50%, at 41.85% and 42.43% for 150 ng/mL and 750 ng/mL, respectively. Additionally, the extraction recovery of carbamazepine was relatively low (54.2 and 53.92 ng/mL). Both compounds showed a higher degree of interaction with the sorbent in a more alkaline environment. However, the aim of the research was to develop a method that allowed the simultaneous determination of all twelve compounds, which was achieved at the expense of low extraction efficiency of those two analytes (Table 1).

### 2.5. Stability

The stability of the analytes was determined in both biological materials and extracts during their presence in the autosampler. Data on the stability of the analytes are summarized in Table 2, in which the analyte concentrations are expressed as a percentage of the analyte content determined on the first day of the analysis. Based on the results of the study, it can be concluded that the compounds stored in the refrigerator at 8 °C were stable. In addition, no degradation was observed during storage at −21 °C. However, when stored at 15 °C in the form of extracts, N-demethyl olanzapine, carbamazepine-10,11 epoxide, and olanzapine decomposed slightly. The highest losses were observed in the case of N-demethyl olanzapine, whose content decreased to approximately 77%.

### 2.6. Comparison of Obtained Results with Literature Data

A summary of the literature data on the quantitation of the most commonly used neuroleptics in saliva and other biological materials is presented in Table 3. Detailed inspection of these data revealed that both the newly developed method and methods reported in the literature are characterized by *R*^2^ > 0.99. In addition, other psychoactive drugs (antidepressants, benzodiazepines, and antiepileptic drugs) were tested during the same procedure, but to date, analytes and their metabolites in saliva had not been determined simultaneously, as indicated in Table 3. Frequently, the analysis was carried out using liquid chromatography coupled with MS detection. Due to the high sensitivity, LC-MS allows an LOQ of several ng/mL [8,9,16,17,18,19,20,21,22,23] and in some cases below 1 ng/mL [12,13,24,25,26,27]. In the analysis of psychoactive compounds among other neuroleptics using HPLC with UV or DAD detection, the LOQ was much worse, at the level of several or even 20 ng/mL [4,11,28]. The exceptions were quetiapine and risperidone, for which LOQ was determined at 9 and 2 ng/mL, respectively. The LOQ of the newly developed method was assumed at 10 ng for all compounds.

The simultaneous determination of many analytes reduces the time of analysis and amount of sample consumed. In several cases, methods have been developed that allow the simultaneous determination of neuroleptics together with their metabolites. The analysis was carried out in serum and saliva, and LLE was used to isolate the compounds [16,17,18,19,20,21]. However, LLE requires the use of more toxic and volatile solvents. Solid-phase extraction allows reduced consumption of such solvents but was used only for the determination of parent compounds in saliva and serum [28]. In contrast, μ-SPE and protein precipitation were performed for the analysis of five neuroleptics and their two metabolites in plasma [24].

### 2.7. Clinical Application

**Figure 3:**
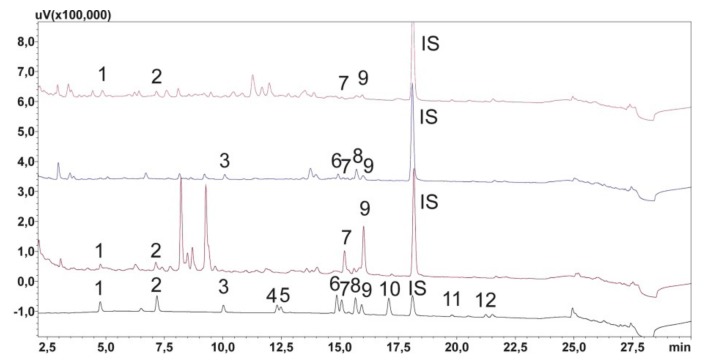
Chromatogram of saliva extracts of patients treated with studied compounds: a—quetiapine and olanzapine; b—clozapine, risperidone, and quetiapine; c—quetiapine and olanzapine; d—chromatogram of calibration solutions. 1—N-demethyl olanzapine; 2—olanzapine; 3—9-OH-risperidone; 4—risperidone; 5—carbamazepine-10,11 epoxide; 6—N-desmethylclozapine; 7—norquetiapine; 8—clozapine; 9—quetiapine; 10—carbamazepine; 11—IS (chlordiazepoxide); 12—dehydroaripiprazole; 13—aripiprazole.

Forty (40) saliva samples from patients with mental disorders were subjected to chromatographic analysis. The developed method allowed the detection and determination of test compounds in all analyzed samples. Examples of the chromatograms of saliva sample extracts derived from the patients are shown in Figure 3. The study shows that the concentrations of risperidone and its metabolite in the saliva of patients were in the ranges 14.43–172.11 ng/mL and 23.87–142.33 ng/mL, respectively, which is consistent with the data in the literature [10,12,14,15]. There are no unequivocal literature data on the concentration of clozapine in saliva. Earlier publications reported that the concentrations of clozapine and its metabolite in saliva were in the ranges 40–364 ng/mL and 40–245 ng/mL, respectively [11]. These results only partially coincided with those of our study, i.e., the concentrations of the parent drug and its metabolite were found to be 40.29–930.97 ng/mL and 20.94–928.36 ng/mL, respectively. Higher values of marked substances may be due to the fact of longer treatments, as demonstrated in other studies [31]. Their authors noticed that in patients starting treatment with clozapine, its concentration in the saliva was usually low and did not exceed 510 ng/mL. However, patients treated for more than two years had drug concentrations of 550–920 ng/mL [31]. Another analyte in our study was aripiprazole, whose highest concentration in the saliva of patients was 19.01 ng/mL, which is consistent with the data in the literature [10]. The quetiapine level (29.21–791.04 ng/mL) was also consistent with the data in the literature [10]. There is no information in the literature on the concentration of norquetiapine in the saliva of people treated with quetiapine. Detailed data on the concentrations of the analytes and their metabolites in the tested saliva samples are summarized in Table 4.

## 3. Materials and Methods

### 3.1. Reagents and Solutions

Acetonitrile, methanol, FA, and ammonia were purchased from POCh (Gliwice, Poland). Trimethylamine (TEA) was obtained from Sigma–Aldrich (St. Louis, MO, USA). All reagents were of HPLC super-grade purity. Deionized water was purified by ultra-Toc/UV Hydrolab (Straszyn, Poland). Standards for aripiprazole, quetiapine, olanzapine, and carbamazepine came from Polpharma (Starogard Gdański, Poland). Dehydroaripiprazole, carbamazepine-10,11 epoxide solution (1 mg/mL), risperidone solution (1 mg/mL), 9-OH-risperidone solution (1 mg/mL), clozapine solution (1 mg/mL), N-desmethylclozapine solution (1 mg/mL), and norquetiapine solution (1 mg/mL) were obtained from Sigma–Aldrich (St. Louis, MO, USA). N-demethyl olanzapine was purchased from Santa Cruz Biotechnology (Dallas, TX, USA). The internal standard (IS), chlordiazepoxide, was purchased from Polfa Tarchomin (Warsaw, Poland).

Standard solutions of aripiprazole, quetiapine, olanzapine, and carbamazepine were prepared by dissolving 10 mg of the substance in 10 mL of methanol. Working solutions at a concentration of 10 μg/mL were prepared by diluting 40 μL of analyte stock solutions (1 mg/mL) with methanol to a volume of 4 mL. A 1 μg/mL working solution was prepared by mixing 40 μL of basic analytes (100 μg/mL) with methanol to a volume of 4 mL. The chlordiazepoxide (IS) standard solution at a concentration of 1 mg/mL was prepared by dissolving 10 mg of the substance in 10 mL of methanol. The working solution was made by diluting the basic solution in methanol. All stock and working standard solutions were stored at −21 °C.

### 3.2. Collection and Pretreatment of Saliva Samples

Saliva samples were taken with Salivettes or by direct placement of saliva in a plastic tube. Prior to collection, volunteers were required to refrain from orally consuming food for at least half an hour and to rinse their mouths with water 10 min before sampling. After being placed in the mouth, Salivettes were chewed for 2 min and then centrifuged at 8000 rpm for 5 min. The resulting saliva was frozen and stored at −21 °C until analysis.

### 3.3. Sample Preparation

The thawed saliva samples were centrifuged, and then 1 mL of fluid was withdrawn and transferred to polypropylene tubes. Next, 1 mL of 2% FA, 1 mL of methanol/water (1:1), an appropriate amount of analyte working solution, and 50 µL of IS solution (10 μg/mL) were added. The samples were then shaken for 20 min and centrifuged at 8000 rpm. The supernatant was loaded onto activated Strata-X-C columns (2 mL of methanol and 2 mL of water). The cartridge was washed with 2 mL of water and 2 mL of a mixture of water and methanol (1:1) and then dried for 10 min. The adsorbed analytes were eluted using a 5% solution of ammonia in methanol. The eluate was dried at 37 °C by nitrogen flux, and the dry residue was dissolved in 100 μL of the mobile phase at 90:10 (*v*/*v*) solvent A to solvent B.

### 3.4. Chromatographic Analysis

Chromatographic separation was carried out with a UHPLC Nexera XR liquid chromatograph (Shimadz, Kyoto, Japan) equipped with an LC-30AD pump, CTO-20AC thermostat, CBM-20Alite control system, SIL-30AC autosampler, UV-VIS SPD-M30A detector with matrix diode and high-sensitivity measuring cell SPD-M30A (85 mm).

Ten-microliter samples were injected onto a Luna Omega 3 μm column (LC Column 150 × 3.0 mm) with Polar C18 100 filling and a precolumn (Polar C18, 4 × 2.0 mm ID). The analytes were eluted at 35 °C, and the mobile phase flow was 0.6 mL/min. The mobile phase was formed by a binary system consisting of water with FA (pH 3.5) with the addition of 0.1% TEA (solvent A) and acetonitrile (solvent B), with a gradient program starting from 85:15 (*v*/*v*) solvent A to solvent B up to 35:65 (*v*/*v*) solvent A to solvent B.

### 3.5. Method Validation

#### 3.5.1. Linearity

**Figure 4:**
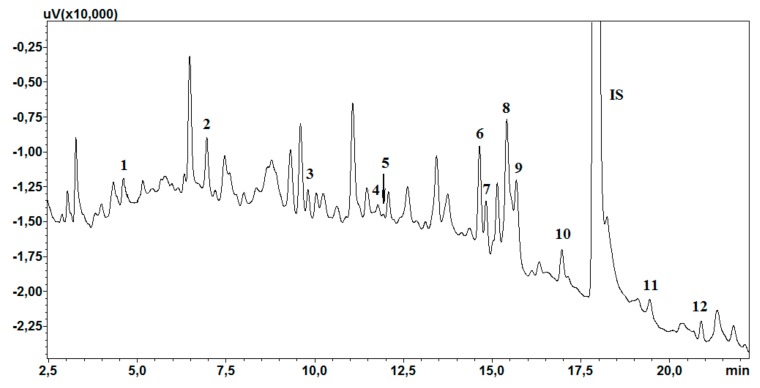
Chromatogram of saliva sample extracts spiked with 10 ng/mL. 1—N-demethyl olanzapine; 2—olanzapine; 3—9-OH-risperidone; 4—risperidone; 5—carbamazepine-10,11 epoxide; 6—N-desmethylclozapine; 7—norquetiapine; 8—clozapine; 9—quetiapine; 10—carbamazepine; IS—chlordiazepoxide; 11—dehydroaripiprazole; 12—aripiprazole.

The linearity of the method was determined by preparing four calibration curves on four consecutive days. The calibration curves were made with appropriate amounts of working solutions added to 1 mL of saliva, for final analyte concentrations of 10, 20, 50, 100, 300, 500, and 1000 ng/mL, and with 50 µL of the IS solution, for a final concentration of 500 ng/mL. The acceptability criteria included a coefficient of determination (*R*^2^) ≥ 0.99 and residuals ≤ 15% at each concentration level. A chromatogram of the lowest concentration of the calibration curve is presented in Figure 4.

#### 3.5.2. Selectivity

**Figure 5:**
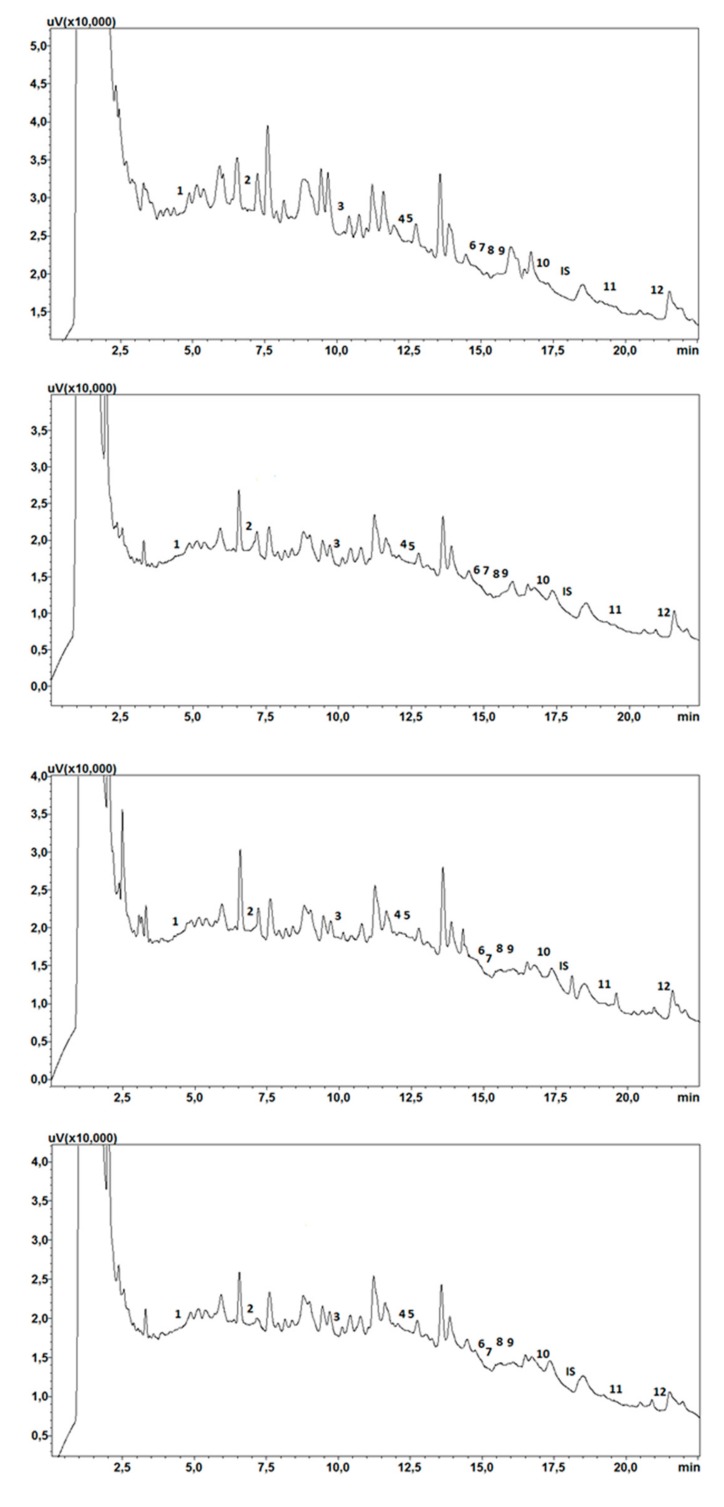
Chromatogram of saliva sample extracts obtained from 4 healthy donors. 1—N-demethyl olanzapine; 2—olanzapine; 3—9-OH-risperidone; 4—risperidone; 5—carbamazepine-10,11 epoxide; 6—N-desmethylclozapine; 7—norquetiapine; 8—clozapine; 9—quetiapine; 10—carbamazepine; IS—chlordiazepoxide; 11—dehydroaripiprazole; 12—aripiprazole.

The selectivity of the method was determined by analyzing 10 blank saliva samples from 10 healthy volunteers. The test was performed to detect interferences caused by endogenous compounds. In the absence of interference, the method was defined as selective (Figure 5).

#### 3.5.3. Intra- and Inter-Day Precision

Intra- and inter-day precision were determined by preparing five samples for three concentrations (15 ng/mL—low QC, 150 ng/mL—medium QC, and 750 ng/mL—high QC). In the case of intra-day precision, five analyses were carried out over one day. The inter-day precision was determined by performing five analyses over four consecutive days (*n* = 20). The precision of the method was expressed using the coefficient of variation (%CV), assuming an acceptable value ≤ 15%.

#### 3.5.4. Limits of Quantification

The limit of quantification (LOQ) means the lowest concentration of the analyte whose signal is ten times higher than the background signal and that can be determined with a precision of RSD < 20% and analyte recovery ±20%. The LOQ of each analyte was determined based on five replications.

#### 3.5.5. Absolute Recovery and Extraction Recovery

Absolute recovery and extraction recovery were determined for two analyte concentrations (150 ng/mL—medium QC and 750 ng/mL—high QC). For each concentration, six samples of saliva were prepared with the appropriate amount of analyte mixture and IS, then subjected to extraction. To determine absolute recovery, the analyte peaks were compared to the peak areas obtained during the analysis of six neat standards of each concentration. The extraction recovery was determined by comparing the surface areas of the extracted analytes with the results obtained during the analysis of each concentration of 10 blank saliva samples to which appropriate amounts of the mixture were added after extraction. The average of six neat standards of each concentration was assumed to be 100%, while an acceptable result was considered to be the one for which the analyte’s value exceeded 50%.

#### 3.5.6. Stability

The stability of the analytes was determined for three concentrations (low QC, medium QC, and high QC). Five samples were prepared at each concentration. The stability of the analytes was tested both in the matrix, for which the samples were stored at +8 °C, and in the freeze–thaw test, for which the samples of saliva were stored at −21 °C. For this purpose, 4 mL of thawed and centrifuged saliva was placed in plastic tubes, and an appropriate amount of the analyte standard mixture solution was added and mixed. Then, 1 mL of sample was subjected to extraction and chromatographic analysis. The remaining volume was frozen or placed in a refrigerator and analyzed in the following days.

The stability of the analytes was also examined during storage of the extracts in the autosampler at 15 °C for 72 h. After this time, the samples were reinjected and chromatographically analyzed alongside a freshly prepared calibration curve.

The compounds whose concentration decreased by less than 15% under the given storage conditions were considered to be stable.

### 3.6. Clinical Application

The usefulness of the developed method for clinical trials was determined by analyzing 40 saliva samples obtained from patients treated with the studied substances in monotherapy alike in polypragmasy. Saliva samples were collected from the patients of the Nervous and Mentally Ill Hospital in Starogard Gdański and the Nursing Home in Damaszka. Saliva samples were collected in the morning at approximately 10:00, without stimulation, by direct placement of the saliva in a plastic test tube. The plastic tubes were tested for possible adhesion of the analytes on their surface. Recovery was determined for two concentrations of analytes (100 and 500 ng/mL) after sample storage at temperatures 8 °C and −21 °C. The results obtained indicated that there was no adhesion on the walls of the plastic tubes used to collect patient saliva samples. The detailed data are provided in Table 5. The test material was then frozen. Before the analysis, the samples were thawed and centrifuged. Then, 1 mL of fluid was analyzed using the procedure described in Section 2.3. Due to the polypragmasy used during sampling, the time that had passed since the administration of individual drugs was not taken into account.

## 4. Conclusions

The presented method enabled the simultaneous determination of five neuroleptics (olanzapine, risperidone, clozapine, quetiapine, and aripiprazole) and the antiepileptic drug carbamazepine together with their metabolites (N-desmethylclozapine, norquetiapine, N-demethyl olanzapine, 9-OH-risperidone, dehydroaripiprazole, and carbamazepine-10,11 epoxide). The SPE enabled the isolation of all test compounds from a small volume of biological material (1 mL). The available data in the literature show that this study was the first use of this extraction method to isolate the tested psychotropic substances and their metabolites from saliva. The developed method is characterized by good linearity, reflected by *R*^2^ > 0.99 for all analytes. Low detection limits of up to 10 ng/mL were achieved. In addition, the CV% for intra-day and inter-day precision did not exceed 15% for any of the studied substances.

The developed method can be used to monitor the levels of neuroleptic drugs and to control their intake by patients by examining readily available biological material such as saliva. The usefulness of the method was confirmed by analyzing 40 saliva samples from patients treated with the studied neuroleptics and carbamazepine. In all cases, we accurately identified the tested active substances.

## Figures and Tables

**Table 1:** Calibration curves (linearity in the range 10–1000 ng/mL) and validation parameters for the developed procedures.

Analyte	*R* ^2^	Slope	Intercept	Intra-Day CV%			Inter-Day CV%			Extraction Recovery (%)		Absolute Recovery (%)	
				15	150	750	15	150	750	150	750	150	750
Aripiprazole	0.9965	0.0011	0.0113	7.50	10.90	6.80	14.16	10.89	6.89	79.25	71.13	60.09	58.32
Dehydroaripiprazole	0.9972	0.0013	0.0150	11.43	10.60	5.35	14.21	10.39	6.99	73.84	73.09	54.13	51.16
Carbamazepine	0.9987	0.0004	0.0014	11.87	6.66	5.48	14.97	8.48	7.02	54.27	53.92	49.18	46.82
Carbamazepine-10,11 epoxide	0.9972	0.00002	0.0002	12.58	12.11	5.77	14.13	12.75	9.60	41.85	42.43	41.40	41.72
Clozapine	0.9983	0.0014	0.0117	5.40	7.59	5.96	14.43	8.16	8.15	86.05	89.55	66.30	54.97
N-desmethylclozapine	0.9994	0.0011	0.0137	5.74	6.14	5.57	10.10	9.42	6.85	84.51	82.04	52.45	60.39
Olanzapine	0.9948	0.0012	0.0214	2.47	9.90	2.33	17.27	9.74	7.49	77.94	91.01	71.61	56.10
N-demethyl olanzapine	0.9963	0.0006	0.0103	2.01	6.76	6.30	13.93	9.82	7.68	75.73	90.62	64.84	56.00
Quetiapine	0.9985	0.0008	0.0167	5.88	5.15	3.91	13.93	8.67	5.35	69.50	67.71	60.404	57.03
Norquetiapine	0.9991	0.0009	0.0115	4.98	6.28	7.25	14.60	10.22	8.22	83.93	81.64	55.27	60.45
Risperidone	0.9990	0.0006	0.0089	9.91	6.98	4.12	10.70	7.04	7.59	87.69	95.66	65.82	59.62
9-OH-risperidone	0.9990	0.0005	0.0108	4.81	3.53	5.46	4.82	7.63	6.97	85.27	92.73	61.95	57.55

**Table 2:** Stability of analytes in spiked saliva stored in a refrigerator at 8 °C and after freeze–thaw cycles at −21 °C. The stability of the extracts was maintained in an autosampler at 15 °C. The values in the table reflect the analyte concentrations as a percentage of the amount determined on the first day of the analysis.

Analyte	−21 °C			8 °C			15 °C		
	15	150	750	15	150	750	15	150	750
Aripiprazole	104.56	105.50	100.56	102.06	104.37	98.19	100.77	105.81	107.05
Dehydroaripiprazole	96.62	104.47	103.63	102.22	100.81	98.41	94.48	98.94	94.15
Carbamazepine	97.46	100.24	98.43	98.67	93.01	97.17	105.11	102.30	101.63
Carbamazepine-10,11 epoxide	97.85	106.34	104.95	94.37	102.88	106.16	83.44	84.15	80.95
Clozapine	103.71	102.87	90.42	102.45	95.16	107.22	87.59	91.13	103.52
N-desmethylclozapine	104.04	107.16	88.85	100.04	93.44	86.48	102.61	104.16	110.63
Olanzapine	104.41	95.73	94.50	92.28	108.57	84.63	98.22	80.95	81.17
N-demethyl olanzapine	98.62	99.03	95.98	104.78	102.72	93.67	76.43	77.71	78.25
Quetiapine	94.10	95.38	89.75	95.55	94.05	103.32	102.39	109.49	112.65
Norquetiapine	104.79	110.72	94.04	108.10	112.46	105.59	106.99	106.27	109.07
Risperidone	100.32	112.60	90.87	98.71	103.92	108.15	101.45	106.90	103.88
9-OH-risperidone	103.89	112.47	90.32	106.80	102.79	107.64	105.16	99.07	98.29

**Table 3:** A summary of current literature on neuroleptic analysis in saliva and other biological material.

Biological Matrices	Analytes	Isolation	Separation and Detection	LOQ	Linearity	*R* ^2^	Reference
Saliva	carbamazepine	LLE	HPLC-UV	0.02 µg/mL	0.02–3.0 µg/mL	0.99997	[4]
Saliva, serum	carbamazepine, phenytoin, phenobarbital	proteins precipitation	HPLC-UV	-	0.5–100 μg/L	-	[5]
Saliva, serum, plasma	aripiprazole, citalopram (escitalopram), DM-citalopram (DM-escitalopram), duloxetine, mirtazapine, pipamperone, pregabalin, promethazine, quetiapine, venlafaxine, DM-venlafaxine	proteins precipitation	LC-MS/MS	1 ng/mL (aripiprazole) 2 ng/mL (pipamperone) 2 ng/mL (quetiapine)	18.6–661 ng/mL (aripiprazole) 13.9–528 ng/mL (pipamperone) 29.2–581 ng/mL (quetiapine)	>0.99	[8]
Saliva, blood	venlafaxine, O-desmethylvenlafaxine quetiapine, citalopram	-	LC-MS/MS, HPLC-UV (citalopram)	1 ng/mL (quetiapine)	-	-	[9]
Plasma, whole blood, oral fluid	amisulpride, aripiprazole, clozapine, quetiapine, risperidone, sulpiride	LLE	LC-MS/MS	10 µg/L (clozapine, norclozapine) 5 µg/L (aripiprazole dehydroaripiprazole) 2 µg/L (quetiapine) 1 µg/L (risperidone, 9-hydroxyrisperidone)	-	>0.99	[10]
Saliva, plasma	clozapine, desmethylclozapine	LLE	LC-UV	15 ng/mL	100–1000 ng/mL	0·9990	[11]
Plasma, saliva	risperidone, 9-hydroxyrisperidone	proteins precipitation	LC-MS/MS	250 pg/mL	1–100 ng/mL	>0.99	[12]
Plasma, saliva	risperidone, 9-hydroxyrisperidone	SPE	LC-MS/MS	0.2 ng/mL	0.2–100-ng/mL (plasma) 0.4–200 ng (saliva)	-	[13]
Plasma, saliva	risperidone, 9-hydroxyrisperidone	MEPS	HPLC-coulometric detection	0.5 ng/mL (risperidone) 0.17 ng/mL (9-hydroxyrisperidone)	0.5–50.0 ng/mL (risperidone) 0.5–100.0 ng/mL (9-hydroxyrisperidone)	>0.999	[14]
Plasma, urine, saliva	risperidone, 9-hydroxyrisperidone	MEPS	LC-UV	2 ng/mL (risperidone) 3 ng/mL (9-hydroxyrisperidone)	2–200 ng/mL (risperidone) 3–300 ng/mL (9-hydroxyrisperidone)	0.9996	[15]
Plasma, serum, oral fluid, hemolyzed whole blood	amisulpride, aripiprazole, dehydroaripiprazole, clozapine, norclozapine, olanzapine, quetiapine, risperidone, 9-hydroxyrisperidone, sulpiride	LLE	LC–MS/MS	1–5 µg/L	10–500 µg/L (aripiprazole, dehydroaripiprazole 10–2000 µg/L (clozapine, norclozapine) 2–200 µg/L (olanzapine, risperidone, 9-hydroxyrisperidone) 10–800 µg/L (quetiapine)	>0.95	[16]
Serum	haloperidol, reduced haloperidol, iloperidone, hydroxy iloperidone, asenapine, bromperidol, 7-hydroxy quetiapine, 7-hydroxy N-desalkyl quetiapine, N-demethylolanzapine, risperidone, zuclopenthixol, paliperidone, olanzapine, sertindole, lurasidone, pipamperone, dehydro-aripiprazole, amisulpride, N-demethylclozapine, quetiapine, aripiprazole, clozapine, (Levo)sulpiride	LLE	UHPLC–MS/MS	0.1–5 ng/mL	1–100 ng/mL (7-hydroxy N-desalkyl quetiapine, N-demethylolanzapine) 1–150 ng/mL (risperidone) 1–300 ng/mL (paliperidone, olanzapine) 10–1000 ng/mL (dehydro-aripiprazole) 10–1500 ng/mL (N-demethylclozapine, quetiapine, aripiprazole, clozapine)	>0.99	[20]
Oral fluid, serum	amisulpride, aripiprazole, bromperidol, clozapine, N-desmethylclozapine, haloperidol, reduced haloperidol, olanzapine, N-desmethylolanzapine, paliperidone, pipamperone, quetiapine, 7OH-N-desalkyl-quetiapine, 7OH-quetiapine, risperidone, zuclopenthixol	LLE	UHPLC-MS/MS	0.16–16 ng/mL	6.40–2400 ng/mL (aripiprazole) 16.00–2400 (clozapine, N-desmethylclozapine, quetiapine) 1.6–480 ng/mL (olanzapine) 1.6–160 ng/mL (N-desmethylolanzapine, 7OH-N-desalkyl-quetiapine, 7OH-quetiapine) 0.32–480 ng/mL (paliperidone) 0.32–240 ng/mL (risperidone)	>0.99	[21]
Saliva, serum	risperidone, citalopram, clozapine, quetiapine, levomepromazine, perazine, aripiprazole	SPE	HPLC-DAD HPLC-MS	13.35 ng/mL (risperidone) 18.83 ng/mL (clozapine) 9.24 ng/mL (quetiapine) 16.58 ng/mL (aripiprazole)	10–1000 ng/mL	>0.999	[28]
Plasma, oral fluid	chlorpromazine, clozapine, haloperidol, olanzapine, quetiapine, cyamemazine and, levomepromazine	SPE	GC-MS/MS	1 to 10 ng/mL	2–600 ng/mL (plasma) 2–400 ng/mL (oral fluid)	0.99	[29]
Plasma	aripiprazole, dehydro-aripiprazole, olanzapine, risperidone, paliperidone, quetiapine, clozapine, caffeine	µ-SPEprotein precipitation	LC-MS/MS	0.2–1200 ng/mL	0.18–120 ng/mL (aripiprazole) 0.25–80 ng/mL (dehydro-aripiprazole) 1.00–100 ng/mL (olanzapine) 0.70–60 ng/mL (risperidone) 0.20–30 ng/mL (paliperidone) 0.50–160 ng/mL (quetiapine) 0.50–1000 ng/mL (clozapine)	>0.99	[24]
Plasma	olanzapine	LLE	LC/MS	2.0 ng/mL	2–300 ng/mL	0.9993	[22]
Plasma	olanzapine, N-desmethylolanzapine	protein precipitation	LC-MS/MS	0.2 ng/mL (olanzapine) 0.5 ng/mL (N-desmethylolanzapine)	0.2–120 ng/mL (olanzapine) 0.5–50 ng/mL (N-desmethylolanzapine)	>0.99	[25]
Plasma	olanzapine, quetiapine, clozapine, haloperidol and chlorpromazine, mirtazapine, paroxetine, citalopram, sertraline, imipramine, clomipramine, fluoxetine, carbamazepine lamotrigine, diazepam clonazepam	protein precipitation	LC-MS/MS	0.2–5.0 ng/mL	0.2–40.5 ng/mL (olanzapine) 0.5–510.0 ng/mL (quetiapine) 1.5–1550.0 ng/mL (clozapine) 0.5–10500.0 ng/mL (carbamazepine)	>0.9979	[26]
Plasma	haloperidol, olanzapine, clonazepam, mirtazapine, paroxetine, citalopram, sertraline, chlorpromazine, imipramine, clomipramine, quetiapine, diazepam, fluoxetine, clozapine, carbamazepine, andlamotrigine	MEPS	LC–MS/M	0.05–1.00 ng/mL	0.5–40.50 ng/mL (olanzapine) 10–410 ng/mL (quetiapine) 150–1550 ng/mL (clozapine) 500–10500 ng/mL (carbamazepine)	>0.99	[27]
Serum, urine	clozapine, norclozapine, clozapine-N-oxide	LLE	LC-MS/MS	1.0 ng/mL (serum) 2.0 ng/mL (urine)	1–2000 ng/mL	>0.99	[23]
Plasma	chlorpromazine, haloperidol, cyamemazine, quetiapine, clozapine, olanzapine, levomepromazine	MEPS	GC-MS/MS	0.2–1 ng/mL	1–1000 ng/mL (clozapine) 4–1000 ng/mL (quetiapine) 0.8–200 ng/mL (olanzapine)	>0.99	[30]

LLE - liquid–liquid extraction; SPE - solid-phase extraction; MEPS - microextraction by packed sorbent.

**Table 4:** The concentrations of neuroleptic drugs and their metabolites in saliva samples (drug doses in mg/day used by patients are given in the parentheses).

Saliva Sample	Concentration (ng/mL)/Dose (mg/day)											
	Aripiprazole	Dehydroaripiprazole	Carbamazepine	Carbamazepine-10,11-Epoxid	Clozapine	N-Desmethyl Clozapine	Olanzapine	N-Demethyl Olanzapine	Quetiapine	Norquetiapine	Risperidone	9-OH-Risperidone
1			1051.52 (800)	1534.39			21.32 (15)	31.74	410.14 (700)	25.66		
2									85.82 (300)	73.50	18.33 (3)	57.68
3							78.34 (20)	17.17	317.00 (300)	247.89	29.78 (6)	50.57
4							19.02 (20)	49.71	181.87 (600)	285.91	29.10 (4)	104.68
5			187.65 (400)	35.36			21.09 (20)	26.81	32.60 (300)	12.		
6							68.23 (20)	108.89	675.52 (200)	34.26		
7									456.87 (800)	134.21	172.11 (3)	37.37
8							18.55 (20)	16.65	31.71 (150)	12.97		
9	19.01 (15)	15.53					24.39 (20)	16.17				
10	11.48 (15)	8.62 *					26.02 (20)	18.09				
11									46.68 (150)	15.94		
12					40.29 (150)	20.94			74.55 (200)	15.91	15.33 (6)	78.16
13							30.35 (10)	51.93	733.17 (700)	371.44		
14							18.08 (20)	24.94	51.86 (300)	25.09		
15									77.93 (200)	31.28		
16									68.90 (100)	28.85		
17	10.91 (15)	11.27					24.53 (20)	34.00				
18	16.04 (15)	13.07					24.40 (20)	16.27				
19					930.97 (350)	928.36			791.05 (300)	200.01		
20									74.51 (50)	50.56		
21			132.51 (800)	21.09			17.97 (15)	15.94	54.54 (700)	13.60		
22									48.58 (300)	29.16	14.43 (3)	56.09
23							83.62 (20)	41.45	365.50 (300)	281.07	49.03 (6)	142.33
24							34.11 (20)	19.50	83.21 (600)	139.32	27.70 (4)	23.87
25			155.27 (400)	171.30			33.20 (20)	49.15	131.30 (300)	54.36		
26							70.12 (20)	113.71	55.07 (200)	26.18		
27									68.87 (800)	25.04	17.25 (3)	57.81
28	14.33 (15)	17.97					18.68 (20)	62.00				
29	13.49 (15)	15.40					21.86 (20)	16.40				
30	11.15 (15)	8.11 *					18.32 (20)	17.36				
31									63.81 (150)	16.25		
32					69.63 (150)	45.60			80.56 (200)	22.79	17.05 (6)	103.92
33							37.51 (10)	16.68	440.16 (700)	237.3		
34							17.92 (20)	16.18	115.74 (300)	15.87		
35									60.86 (200)	14.70		
36									29.21 (100)	23.79		
37	15.69 (15)	10.11					21.54 (20)	18.77				
38	11.50 (15)	14.22					20.57 (20)	16.17				
39					495.77 (350)	274.28			226.35 (300)	51.05		
40									51.25 (50)	14.15		
Mean	13.73	12.70	381.74	440.53	384.16	317.29	32.10	33.99	193.07	81.62	39.01	71.25

* The determined concentrations were below the concentrations for which the calibration curve was determined.

**Table 5:** Adhesion of analytes on the plastic tube walls stored in fridge at 8 °C and after freeze at −21 °C. Values in table reflect the percentage of analyte AUC in relation to the AUC determined before the storage.

Analyte	AUC	Recovery (%)		AUC	Recovery (%)	
	100 ng	8 °C	−21 °C	500 ng	8 °C	−21 °C
Aripiprazole	7928	98.87	99.69	95,344	105.63	104.15
Dehydroaripiprazole	8568	100.16	97.90	93,141	101.13	103.79
Carbamazepine	40,166	97.15	98.92	212,738	98.74	100.81
Carbamazepine-10,11 epoxide	6357	98.12	100.20	31,827	99.58	101.62
Clozapine	37,397	97.78	98.19	222,722	103.05	101.34
N-desmethylclozapine	37,580	102.36	98.20	242,767	102.05	105.11
Olanzapine	42,865	99.79	99.43	213,827	101.65	99.91
N-demethyl olanzapine	20,668	96.99	101.35	127,724	101.00	103.58
Quetiapine	21,134	102.79	95.95	115,710	104.01	104.03
Norquetiapine	34,733	101.62	96.77	197,758	104.91	98.19
Risperidone	37,187	97.52	100.81	190,495	103.72	99.90
9-OH-risperidone	15,893	102.79	101.81	83,981	104.51	98.23
